# Inner Ear Morphology in the Atlantic Molly *Poecilia mexicana*—First Detailed Microanatomical Study of the Inner Ear of a Cyprinodontiform Species

**DOI:** 10.1371/journal.pone.0027734

**Published:** 2011-11-15

**Authors:** Tanja Schulz-Mirbach, Martin Heß, Martin Plath

**Affiliations:** 1 Department of Earth and Environmental Sciences, Ludwig-Maximilians-University, Munich, Germany; 2 Department of Biology I, Ludwig-Maximilians-University, Martinsried, Germany; 3 Biologicum, J.W. Goethe-University Frankfurt am Main, Frankfurt am Main, Germany; University of Oldenburg, Germany

## Abstract

**Background:**

Fishes show an amazing diversity in hearing abilities, inner ear structures, and otolith morphology. Inner ear morphology, however, has not yet been investigated in detail in any member of the diverse order Cyprinodontiformes. We, therefore, studied the inner ear of the cyprinodontiform freshwater fish *Poecilia mexicana* by analyzing the position of otoliths *in situ*, investigating the 3D structure of sensory epithelia, and examining the orientation patterns of ciliary bundles of the sensory hair cells, while combining μ-CT analyses, scanning electron microscopy, and immunocytochemical methods. *P. mexicana* occurs in different ecotypes, enabling us to study the intra-specific variability (on a qualitative basis) of fish from regular surface streams, and the Cueva del Azufre, a sulfidic cave in southern Mexico.

**Results:**

The inner ear of *Poecilia mexicana* displays a combination of several remarkable features. The utricle is connected rostrally instead of dorso-rostrally to the saccule, and the macula sacculi, therefore, is very close to the utricle. Moreover, the macula sacculi possesses dorsal and ventral bulges. The two studied ecotypes of *P. mexicana* showed variation mainly in the shape and curvature of the macula lagenae, in the curvature of the macula sacculi, and in the thickness of the otolithic membrane.

**Conclusions:**

Our study for the first time provides detailed insights into the auditory periphery of a cyprinodontiform inner ear and thus serves a basis—especially with regard to the application of 3D techniques—for further research on structure-function relationships of inner ears within the species-rich order Cyprinodontiformes. We suggest that other poeciliid taxa, or even other non-poeciliid cyprinodontiforms, may display similar inner ear morphologies as described here.

## Introduction

Fishes show an amazing diversity in hearing abilities [1], [2], inner ear structures [3]–[6], and otolith morphology [7], [8]. Until now, however, only few studies investigated aspects of inner ear morphology within the diverse order Cyprinodontiformes (e.g., green swordtails, *Xiphophorus hellerii* [9]; guppies, *P. reticulata* [10], both Poeciliidae; redtail splitfin, *Xenotoca eiseni* [3], Goodeidae).

The Central American Atlantic molly, *Poecilia mexicana* (Cyprinodontiformes, Poeciliidae) has been established as a model to study various aspects of local adaptation and ecological speciation [11], [12]. We therefore chose *P. mexicana* for our study of cyprinodontiform inner ear morphology.For the Atlantic molly several populations are described that have adapted to extreme environmental conditions (e.g., cave life [13]–[15]). Considering the amazing degree of local adaptation to divergent habitat types observed in this widely distributed species [11], [12], we investigated the intra-specific variability in inner ear morphology by including three populations belonging to two different ecotypes (cave *vs.* surface habitats). So far, only very limited information exists on intra-specific variation of inner ear morphology in teleost fishes (for otolith shape variation among *P. mexicana* populations see refs [16], [17]).

The inner ear of modern bony fishes (Teleostei) is composed of the upper labyrinth (pars superior) containing the three semicircular canals and the utricle and the lower labyrinth (pars inferior) which is build up by the two end organs, saccule and lagena. In each of the end organs a calcareous biomineralisate (otolith) is connected via an otolithic ‘membrane’ (the OM is actually not a biomembrane, but a fibrillar extracellular pellicle; [18]) to the ciliary bundles of the hair cells extending from the sensory epithelium (macula). According to their position in the inner ear, three different otolith types can be distinguished, namely the otolith of the utricle (lapillus), saccule (sagitta), and lagena (asteriscus). Otoliths can either cover the entire macula or only a part of it [3], [5], [6], [19]; in the latter case, the remaining macula area is covered by the otolithic membrane only. Still, the interrelationships between otolith morphology, and features like otolithic membrane structure, or the structure of the respective sensory epithelium are not yet fully understood [20], [21]. Moreover, there exist only few studies dealing with all three otolithic end organs and their functional interactions (e.g., [22], [23]).

Our study provides the first detailed investigation of a cyprinodontiform inner ear while combining an array of different methodological approaches (μ-CT analyses, scanning electron microscopy, and confocal laser scanning microscopy after immunocytochemical staining). Specifically, we focused on the position of otoliths *in situ*, the three-dimensional structure of sensory epithelia, and orientation patterns of ciliary bundles of the sensory hair cells. The primary aim of our study was to provide a basis for further investigations with regard to structure-function relationships of inner ears of cyprinodontiform fishes. In addition, by comparing cave and surface forms of *P. mexicana* (on a qualitative basis) we made a first attempt to describe intra-specific variability and to detect potential differences between ecotypes that might be interpreted as adaptations to extreme environmental conditions (toxic hydrogen sulfide coupled with perpetual darkness).

## Results

### Gross inner ear morphology

**Figure 1:**
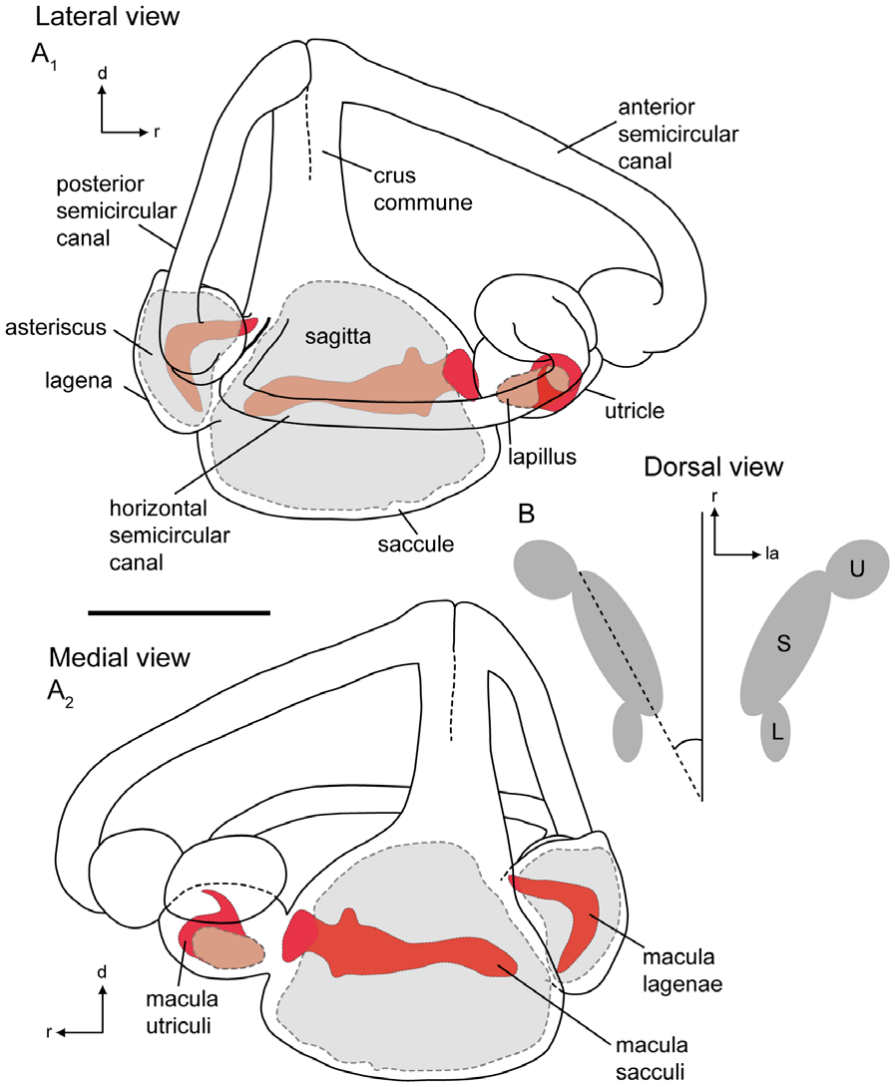
Schematic drawings of the membranous labyrinths of a surface fish of *Poecilia mexicana* from Río Oxolotán. (**A**), right labyrinth with semicircular canals and end organs with otoliths shown in shaded gray and maculae shown in red in lateral view (**A_1_**) and in medial view (**A_2_**). (**B**), left and right end organs in dorsal view displaying the position of end organs with respect to the main axis of the fish. d, dorsal; L, lagena; la, lateral; r, rostral; S, saccule; U, utricle. Scale bar = 1 mm.

**Figure 2:**
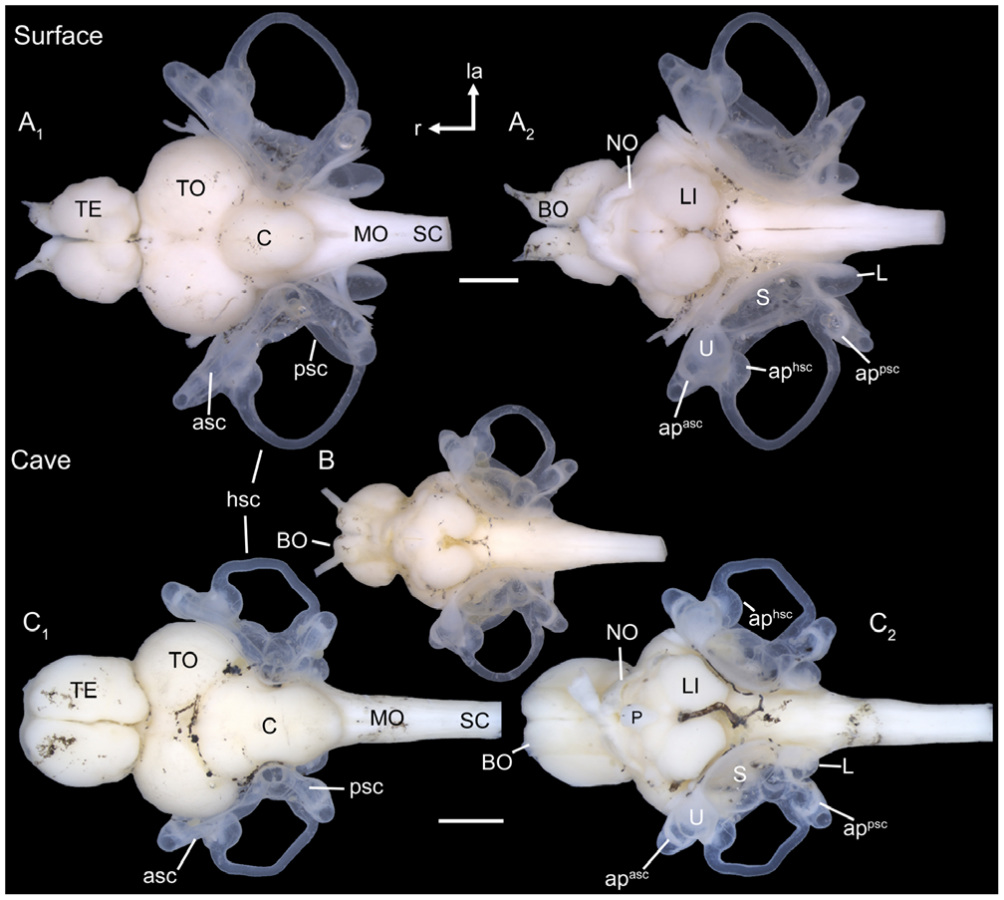
Gross morphology of brain and inner ears. Brain and inner ears of fish from the surface habitat Tampico (**A**) and fish from the Cueva del Azufre (**B, C**). (**A**) female, SL = 54 mm; (**B**) subadult specimen, SL = 26 mm; (**C**) male, SL = 35 mm. (**A_1_, C_1_**): dorsal view; (**A_2_, B, C_2_**): ventral view. TE, telencephalon; C, cerebellum; MO, medulla oblongata; BO, olfactory bulb; TO, optic tectum; P, pituitary gland; NO, optic nerve; SC, spinal cord; LI, inferior lobe; asc, anterior semicircular canal; hsc, horizontal semicircular canal; psc, posterior semicircular canal; ap^asc^, ampulla of the anterior semicircular canal; ap^hsc^, ampulla of the horizontal semicircular canal; ap^psc^, ampulla of the posterior semicircular canal; L, lagena; la, lateral; r, rostral; S, saccule; U, utricle. Scale bars = 1 mm.

The lagena and utricle in *P. mexicana* (*N* = 6) are both smaller than the saccule (Figure 1A–B). The utricle is positioned rostro-laterally to the saccule and a wide opening connects both end organs. The lagena is positioned postero-laterally to the saccule (Figure 1A_2_). The lagena of the surface ecotype shows an almost parallel orientation to the main axis of the fish (Figure 2A_2_), while in the cave ecotype it was found to be more laterally-bent (Figure 2B, C_2_). Moreover, all three semicircular canals end into the saccule (Figure 1A_2_). The caudal end of the horizontal semicircular canal is directly attached to the ampulla of the posterior semicircular canal (Figure 1A_1_).

### Position of otoliths *in situ*

**Figure 3:**
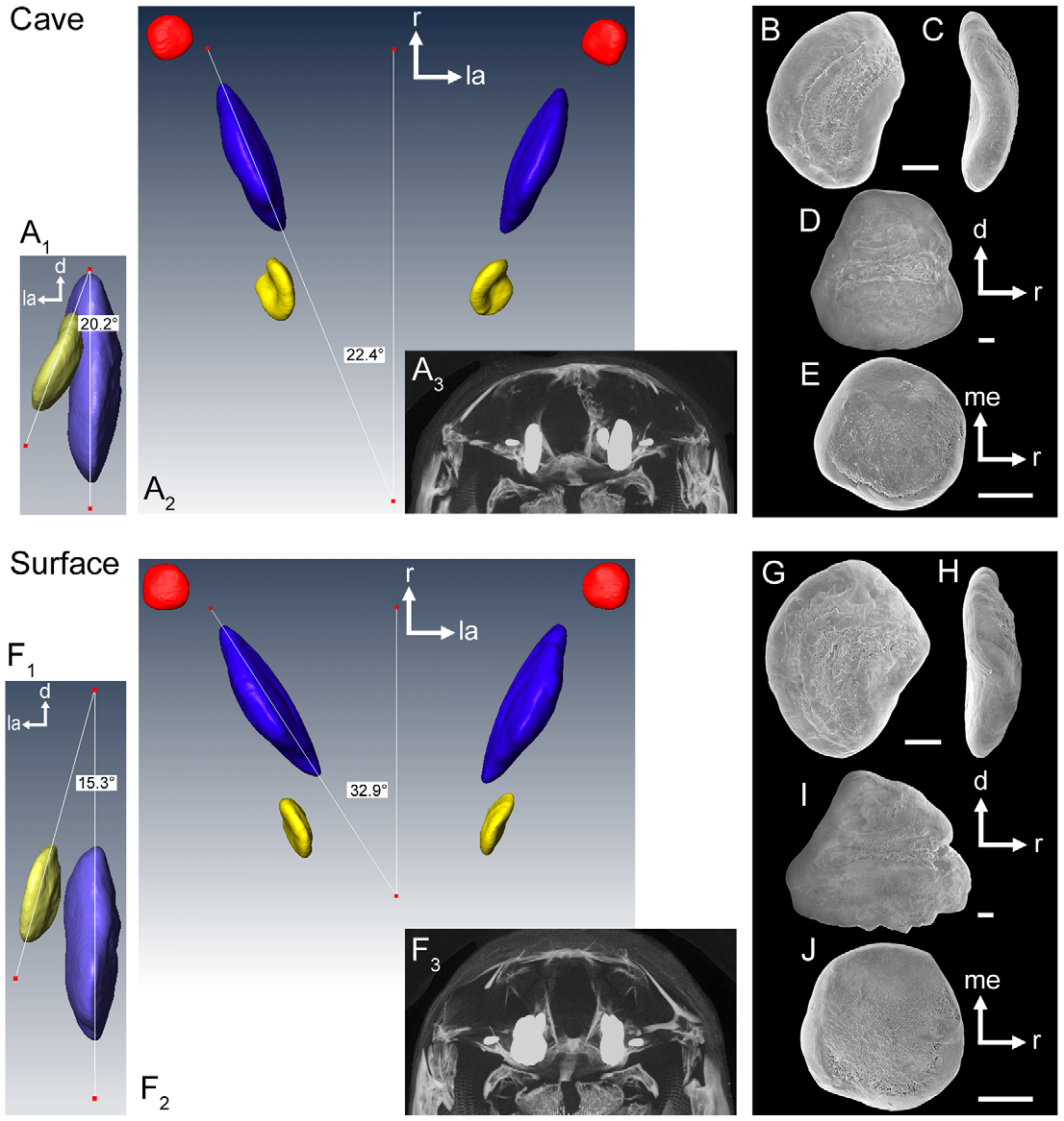
Position of otoliths *in situ*. Three-dimensionally reconstructed otoliths from a cave (SL = 35 mm) and a surface fish (SL = 38 mm, both females) based on μ-CT analyses. In (**A_1_**) and (**F_1_**) the left asteriscus and sagitta are shown in caudal view. (**A_2_**) and (**F_2_**): dorsal view of left and right lapilli (red), sagittae (blue), and asterisci (yellow). (**A_3_**) and (**F_3_**): brightest point projection of μ-CT sections of the neurocranium and otoliths *in situ* shown in rostral view. (**B–E**) and (**G–J**): SEM images of the left otoliths of the cave and the surface fish in medial (**B, D; G, I**), rostral (**C, H**), and ventral views (**E, J**), respectively. B–C, G–H, asterisci; D, I, sagittae; E, J, lapilli. d, dorsal; la, lateral; me, medial; r, rostral. Scale bars = 100 µm.

As typical for most teleost fishes, sagittae (from *N* = 2 individuals) were found to be larger than asterisci and lapilli (Table 1, see also Figure 3 G-J *vs.* B-E; note that otolith size was quantified in a previous study [17] using a much larger sample size, where significant differences among ecotypes were detected). The angle of the sagitta to the main body axis varied between 22° in the examined cave specimen (22° on both sides; Figure 3A_2_) and up to 33° in the surface fish (we determined 33° on the left side and 26° on the right side; Figure 3F_2_). The angle between sagitta and asteriscus was 20° on the left side and 24° on the right side for the cave fish (Figure 3A_1_) and 15° (left side) and 12° (right side), respectively, in surface fish (Figure 3F_1_). The lapillus of cave fish was slightly rotated along the horizontal plane in lateral direction (Figure 3A_2_) compared to the lapillus of surface fish (Figure 3F_2_).

**Table 1:** Volume measurements of the three otolith types based on 3D reconstructions of serial μ-CT scans of a cave (SL = 35 mm) and a surface fish female (SL = 38 mm).

Volume [SL-corrected]	Lapillus (mm^3^) [ratio*100]		Sagitta (mm^3^) [ratio*100]		Asteriscus (mm^3^) [ratio*100]	
Population	left	right	left	right	left	right
Cueva del Azufre, XIII	0.008 [0.023]	0.008 [0.023]	0.175 [0.500]	0.173 [0.494]	0.018 [0.051]	0.017 [0.049]
Río Oxolotán	0.011 [0.029]	0.011 [0.029]	0.217 [0.571]	0.218 [0.574]	0.023 [0.061]	0.023 [0.061]

The numbers in brackets indicate SL-corrected values of the otolith volume [(raw volume measurement/SL)*100].

### Depth of sulcus acusticus

**Figure 4:**
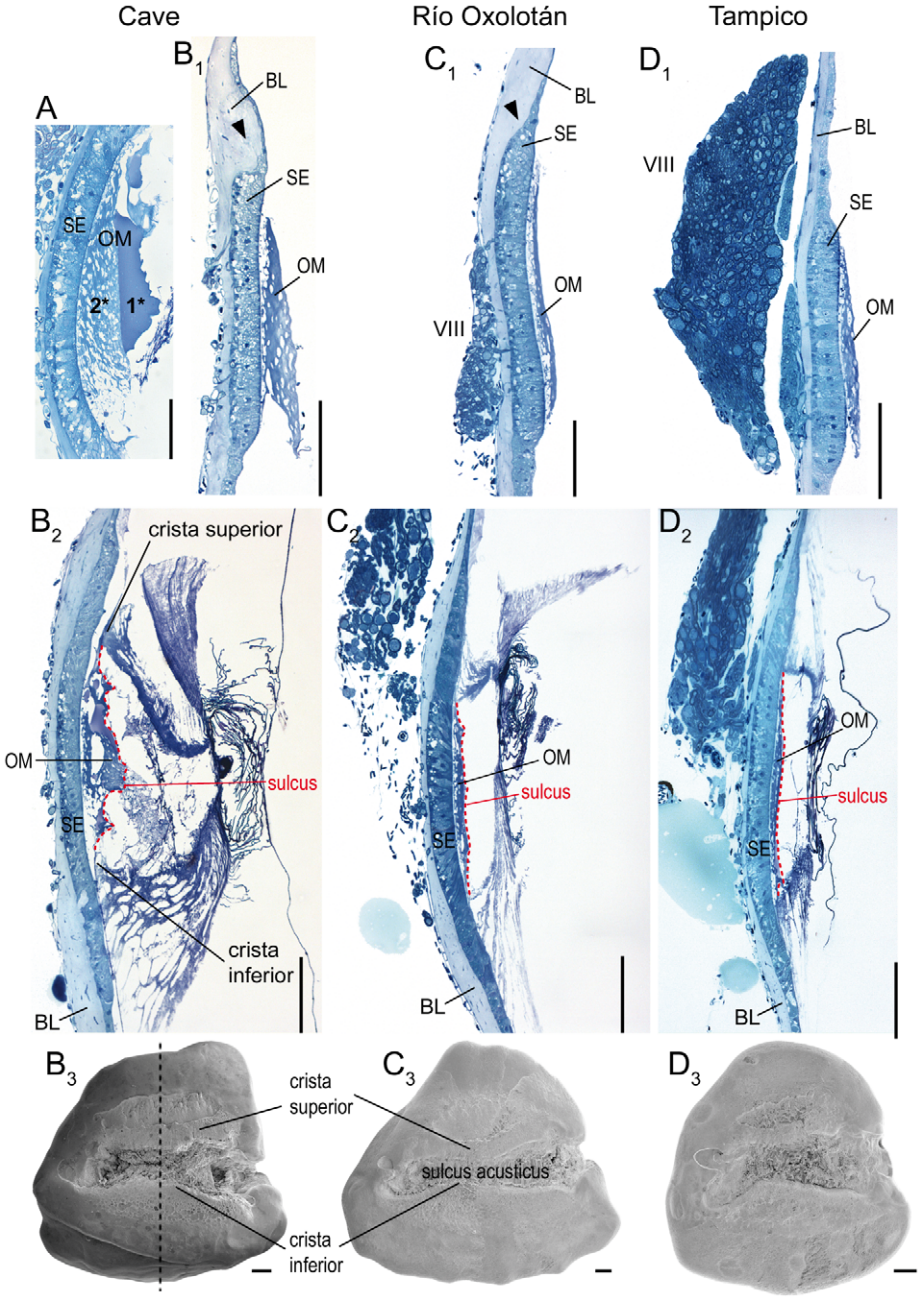
Relationship of the depth of the sulcus acusticus, thickness of the otolithic membrane, and curvature of the macula sacculi. Light micrographs of transversal semithin-sections (thickness: 1 µm) displaying the difference of the thickness of the otolithic membrane between cave (**A, B**) and surface fish (**C, D**). (**A**), cave fish female (SL = 51 mm), (**B**), cave fish male (SL = 37 mm), (**C**), surface fish from Río Oxolotán (female, SL = 44 mm), and (**D**), surface fish from Tampico (female, SL = 30 mm). (**B_1_–D_1_**), sections of the rostral part of the right macula sacculi not overlain by the sagitta. Black arrowheads indicate a dorsal swelling of the basal lamina flanking the macula which is distinctly developed in cave fish and in surface fish from Río Oxolotán. (**A, B_2_–D_2_**), sections in the central region of the sulcus acusticus as indicated by the black dashed line in (**B_3_**). (**B_3_–D_3_**) show the medial face of the left otoliths of the same individuals of which the sections in (**B_1_–D_1_**) and (**B_2_–D_2_**) are presented. 1*, upper ‘compact’ part of the otolithic membrane; 2*, lower part of the otolithic membrane displaying numerous pores. BL, basal lamina; OM, otolithic membrane; SE, sensory epithelium (macula sacculi); VIII, part of the eighth cranial nerve. Sections were stained with Richardson's solution. Scale bars = 100 µm.

The depth of the sulcus acusticus was investigated in *N* = 6 individuals. In fish with thick sagittae and a deep sulcus (mostly observed in cave fish), the otolithic membrane (connecting the sulcus with the sensory epithelium) is distinctly thicker (Figure 4A, B_2_) than in individuals that show thin sagittae and a flat sulcus (Figure 4C_2_, D_2_). The otolithic membrane can be divided in two different parts: a lower, more porous part and an upper, more compact (‘gelatinous’) part (Figure 4A). Sagittae with a flat sulcus are mainly filled with the porous component of the otolithic membrane, whereas the compact upper part is extremely thin (Figure 4C_2_, D_2_). A strongly bulging crista superior (as often seen in cave fish) leads to a distinctly convex macula sacculi (Figure 4A).

### Overall shape of sensory epithelia and orientation patterns of ciliary bundles

#### *Macula lagenae* (*N* = 11)

**Figure 5:**
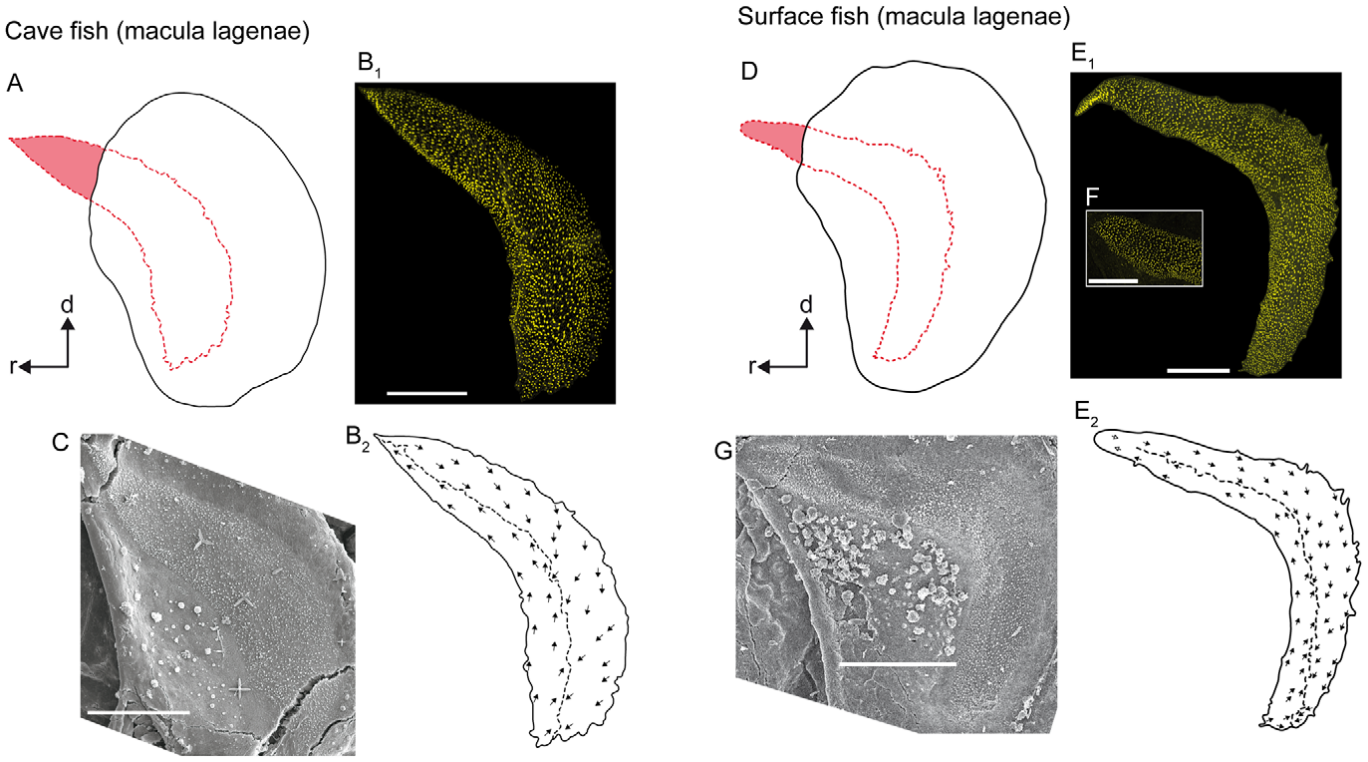
Orientation patterns of ciliary bundles on the macula lagenae. (**A**) and (**D**), drawings of the asterisci and the overlying maculae lagenae showing the amount of the region of the sensory epithelium not overlain by the otolith in a cave fish (**A**, male; SL = 31 mm) and a surface fish from Río Oxolotán (**D**, female; SL = 34 mm). (**B_1_**) the left macula lagenae (mirrored) of the cave fish and (**E_1_**) the right macula lagenae of the surface fish of which the stereocilia of ciliary bundles were stained with TRITC-labelled phalloidin. The macula lagenae in the cave fish (**B_1_**) is only slightly bent while it is boomerang-shaped in the specimen from the surface habitat (**E_1_**). Note that the tip of the rostral arm in (**E_1_**) is slightly inflated. (**F**) shows the shape of an intact tip of the rostral arm of the macula lagenae of another surface fish (female; SL = 40 mm). (**B_2_**) and (**E_2_**), drawings of the same maculae as shown in (**B_1_**) and (**E_1_**), respectively, displaying a similar orientation pattern of the ciliary bundles in cave (**B_2_**) and surface fish (**E_2_**). (**C**) and (**G**), SEM images of the right macula lagenae of another cave fish (male; SL = 30 mm) and of the left macula lagenae (mirrored) of another surface fish (female; SL = 40 mm). d, dorsal, r, rostral. Scale bars = 100 µm.

**Figure 6:**
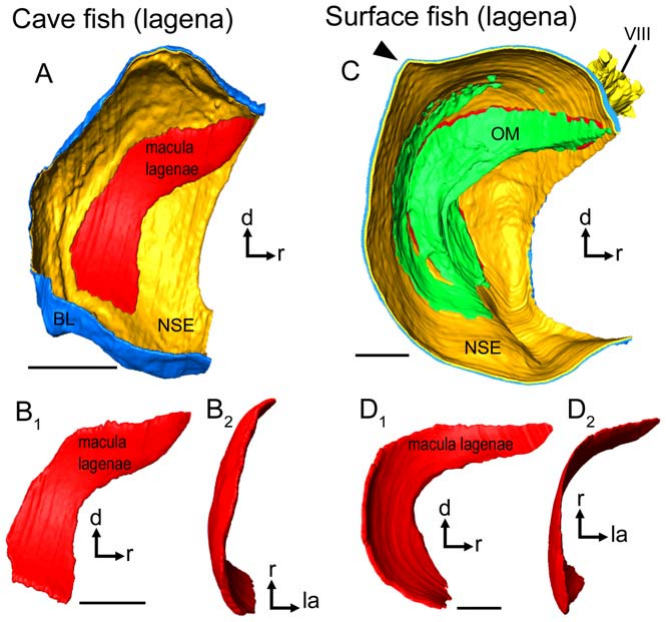
Three-dimensionally reconstructed lagena and macula lagenae of cave fish and surface fish from Tampico. (**A**) and (**C**), lagenae in lateral view showing the macula, (the otolithic membrane), the non-sensory epithelium, and the basal lamina in a cave (**A**; male, SL = 35 mm) and a surface fish (**C**; female, SL = 52 mm). Note that the lagena in the surface fish (**C**) displays a distinct caudo-dorsal ‘edge’ (black arrowhead), while the lagena of the cave fish lacks this “edge” (**A**). This feature corresponds to the prominent posterodorsal edge of asterisci from surface fish (see Figure 5D *vs.* A). (**B**) and (**D**) display differences in curvature and especially shape of the macula lagenae of cave (**B**) and surface fish (**D**). The maculae lagenae are shown in lateral (**B_1_**, **D_1_**) and dorsal view (**B_2_, D_2_**). BL, basal lamina; d, dorsal; NSE, non-sensory epithelium; la, lateral; OM, otolithic membrane; r, rostral; VIII, part of the eighth cranial nerve innervating the lagena. Scale bars = 100 µm.

The macula lagenae almost extends into the saccule. The macula varies in shape from a slightly bent crescent moon (cave fish; Figure 5B_1_, C; Figure 6A, B_1_) to a more boomerang-like contour (surface fish; Figure 5E_1_, G; Figure 6D_1_). This variation in macula shape corresponds to differences in the contour of the fossa acustica observed between the two ecotypes. All cave fish examined (12 females, nine males; material partly reexamined from [17]) showed a widened fossa in the ventral part, whereas surface fish from Río Oxolotán (six females, one male) displayed strongly curved and narrow fossae.

In cave fish, the macula lagenae is distinctly pointed at the rostro-dorsal end, while it is slightly widened in this region in surface fish (Figure 5B_1_ *vs.* F). Between one tenth and one ninth (surface fish; Figure 5D) or between one sixth and one fifth of the rostro-dorsal part of the macula lagenae (cave fish) is not covered by the asteriscus (Figure 5A).

Hair bundle orientation in the maculae lagenae is similar in both ecotypes; caudal ciliary bundles are oriented ventrally, whereas those in the rostral part are oriented dorsally (Figure 5B_2_, E_2_; for number ciliary bundles on the macula see Table 2). Ciliary bundles located near the dividing line tend to point towards this line, especially in the ventral part of the macula.

**Table 2:** Overview of number of ciliary bundles (No. of cb), macula area, and number of ciliary bundles standardized to the area (ratio) of the macula sacculi and macula lagenae of cave (males; *N* = 2) and surface fish (females; *N* = 2).

	SL (mm)	Macula sacculi			Macula lagenae		
Population		No. of cb	Area (µm^2^)	Ratio	No. of cb	Area (µm^2^)	Ratio
Cave	31	3,312	135,678	0.024	2,147	44,757	0.048
	30	3,163	121,957	0.026	2,324	47,282	0.049
Río Oxolotán	34	5,143	200,273	0.026	3,165	60,359	0.052
	41	6,045	196,939	0.031	3,796	98,294	0.039

**Figure 7:**
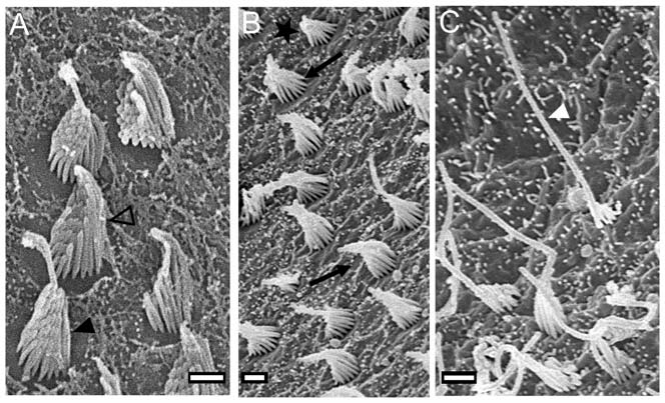
Overview of ciliary bundle types. (**A**), ciliary bundle forming an even slope with a kinocilium only slightly longer than the stereocilia (open arrowhead) (see F3-type in [3]; see also [6]: Figure 5F) and another ciliary bundle with a kinocilium approximately twice as long as the longest stereocilium (black arrowhead) (see [6]: Figure 5E). (**B**), morphological polarization of hair cells: ciliary bundles are oriented in two opposing directions as indicated by black arrows. The star labels a ciliary bundle forming a short and even staircase array similar to the F1-type described in [3]. (**C**), ciliary bundle with a kinocilium at least three times longer than the longest stereocilium (white arrowhead) (see F2-type in [3]; see also [6]: Figure 5A). Scale bars = 1 µm.

The macula lagenae is characterized by a broad margin build up by ciliary bundles with the kinocilium (4–5 µm, rarely up to 8 µm) being at least three times longer than the longest stereocilium (Figure 7C), particularly in the ventral part of the macula. The remaining part of the macula is covered by (i) ciliary bundles with kinocilia (∼3 µm) that are approximately twice as long as the longest stereocilia (Figure 7A) and (ii) ciliary bundles with short kinocilia (1–2 µm), whereby those bundles form an even and shallow staircase array (Figure 7B).

#### *Macula sacculi* (*N* = 11)

**Figure 8:**
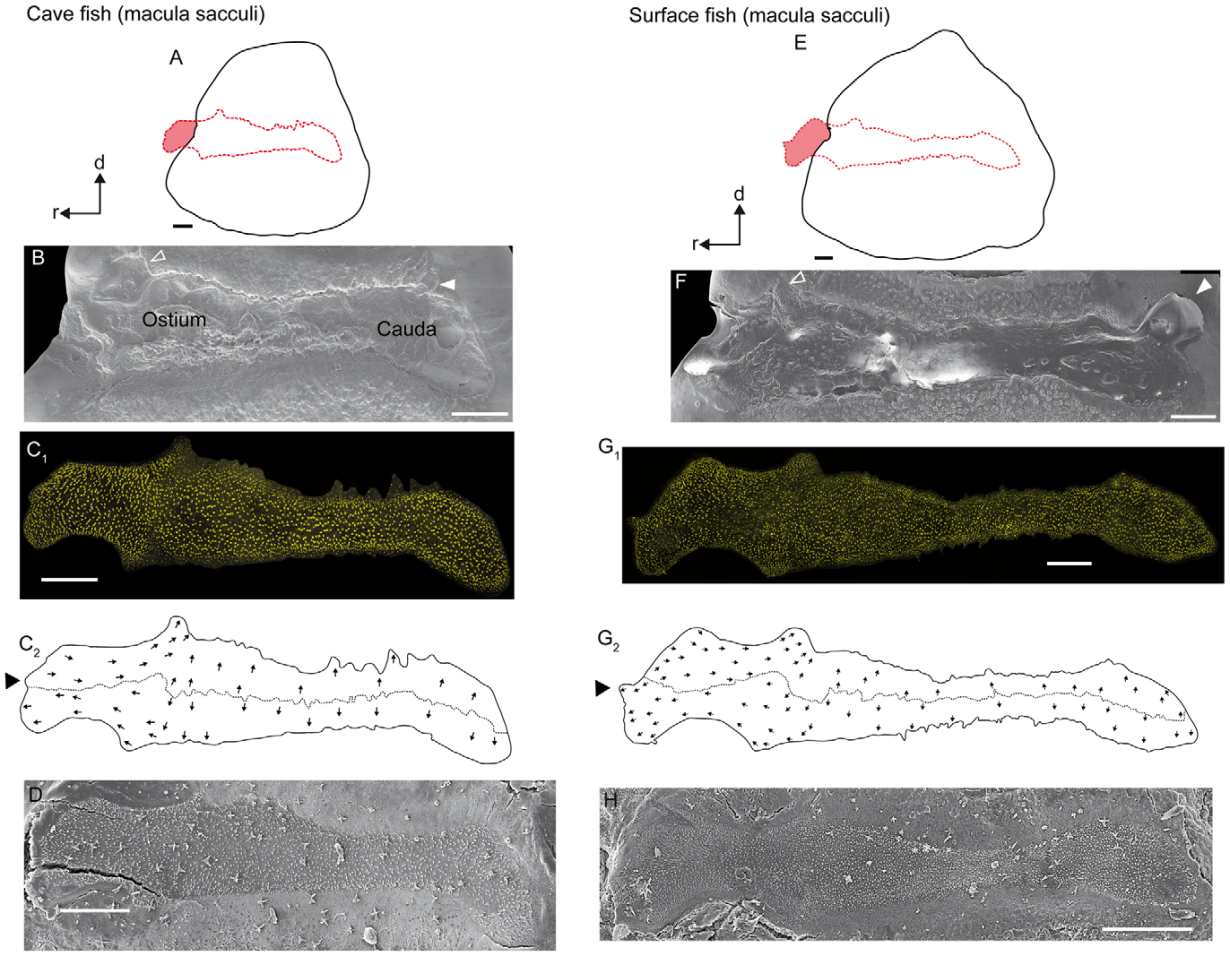
Orientation patterns of ciliary bundles on the macula sacculi. (**A**) and (**E**), drawings of the sagittae and the overlying maculae sacculi showing the amount of the region of the sensory epithelium not overlain by the otolith in a cave (male, SL = 30 mm) and a surface fish from Río Oxolotán (female, SL = 41 mm). (**B**) and (**F**), sulcus acusticus of the corresponding sagitta; the open arrowhead indicates the position of the part of the sulcus housing the dorsal ‘bulge’ of the macula sacculi. The white arrowhead indicates the small caudal sulcus impression rarely seen in sagitta of cave fish (**B**) while this feature is often present in sagittae of surface fish (**F**). The sulcus impression is filled with otolithic membrane (**F**). However, there does not exist a corresponding structure in the macula sacculi (**C_1_**, **G_1_**). (**C_1_**) left macula sacculi (mirrored) of the cave fish and (**G_1_**) right macula sacculi of the surface specimen of which the stereocilia of ciliary bundles were stained with TRITC-labelled phalloidin. (**C_2_**) and (**G_2_**), drawing of the same macula as shown in (**C_1_**) and (**G_1_**) displaying the ‘four quadrant’ orientation pattern of the ciliary bundles. (**D**) and (**H**), SEM images of the right macula sacculi of another cave fish (male; SL = 31 mm) and of the left macula sacculi (mirrored) of another surface fish (female; SL = 40 mm) showing the degree of tissue shrinkage with regard to the maculae in (**C_1_**) and (**G_1_**). d, dorsal, r, rostral. Scale bars = 100 µm.

**Figure 9:**
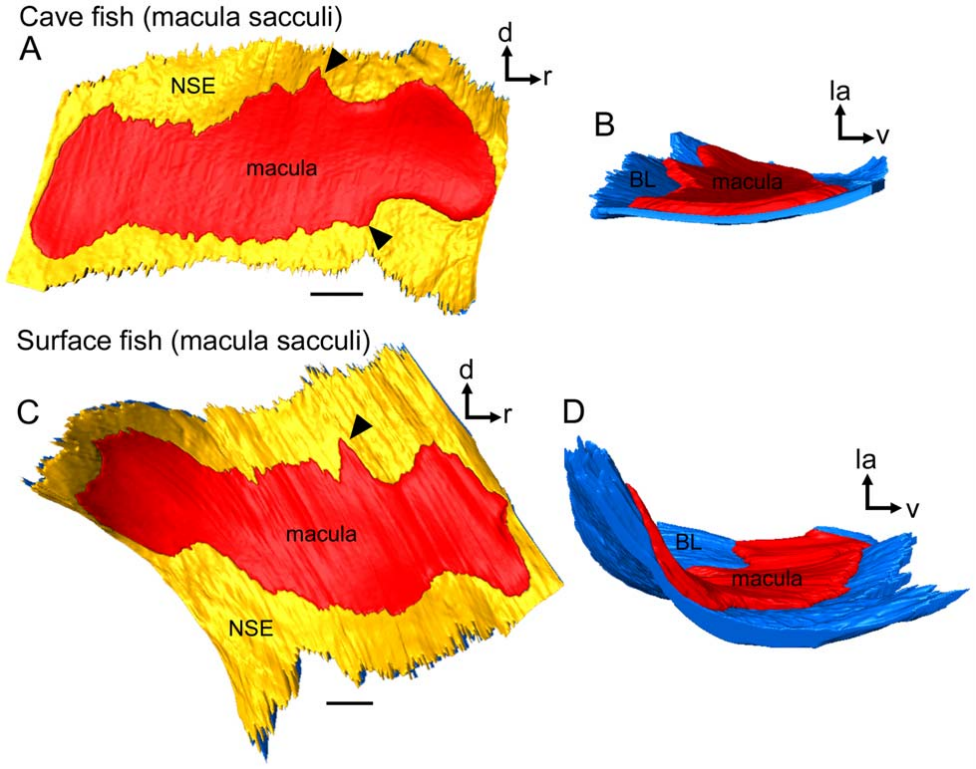
Three-dimensionally reconstructed macula sacculi of cave and surface fish (Tampico). (**A**) and (**C**), maculae sacculi in lateral view showing the macula and the non-sensory epithelium. Black arrowheads label the ventral and dorsal bulges of the macula. (**B**) and (**D**), maculae sacculi in caudal view displaying the different amount of three-dimensional curvature. The strong curvature of the macula sacculi of the surface fish correlates with a thick sagitta whereas the flat almost two-dimensional macula comes along with a rather flat sagitta of the cave fish specimen. BL, basal lamina; d, dorsal; la, lateral; NSE, non-sensory epithelium; r, rostral; v, ventral. Scale bars = 100 µm.

**Figure 10:**
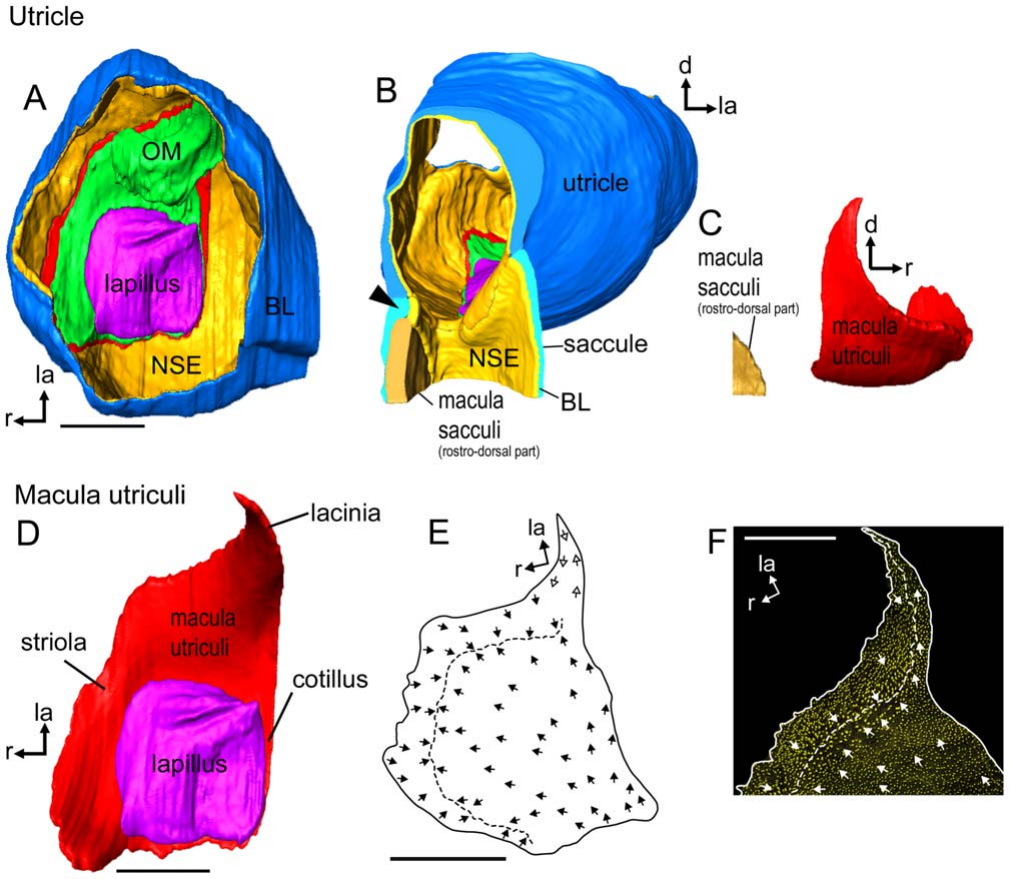
Three-dimensionally reconstructed utricle and orientation pattern of ciliary bundles on the macula utriculi. (**A**), utricle of a cave fish (male, SL = 35 mm) in dorsal view displaying the lapillus, otolithic membrane, macula utriculi, non-sensory epithelium, and the basal lamina. (**B**), Utricle and rostral most part of the saccule in caudal view. The black arrowhead labels the thickened part of the basal lamina that dorsally surrounds the rostral most region of the macula sacculi. (**C**), macula utriculi and the rostral most part of the macula sacculi in lateral view illustrating the closeness of the two maculae to each other. (**D**), macula utriculi and lapillus in dorsal view illustrating the three parts of the macula utriculi, namely the striola, lacinia, and cotillus and the amount of the macula overlain by the otolith. (**E**), Orientation pattern of ciliary bundles on the macula utriculi of a surface fish female (Río Oxolotán, SL = 34 mm) and (**F**), and on the lacinia of the right macula utriculi of a cave fish male (SL = 30 mm). Note that the orientation pattern of the lacinia was inferred from other surface fish individuals. The dashed line in (**E**) and (**F**) shows the change of the orientation pattern with ciliary bundles revealing the opposing orientation of ciliary bundles in the striola region and labels the change of the orientation in the lacinia. BL, basal lamina; d, dorsal; la, lateral; lap, reconstruction based on organic remains of the lapillus; NSE, non-sensory epithelium; me, medial; Msa, macula sacculi; Mu, macula utriculi; OM, otolithic membrane; r, rostral. Scale bars = 100 µm.

The macula sacculi widens caudally and ostially, showing a pronounced dorsal and ventral bulge (Figure 8C_2_, G_2_; Figure 9A, C). The dorsal and ventral bulges fit into corresponding structures of the sulcus acusticus (Figure 8B, F). In some specimens, a third small bulge emerges at the ostial-most part of the macula sacculi (Figure 8C_2_, G_2_). The dorsal and ventral bulges show a distinctly lower density of ciliary bundles in their caudal parts than in the ostial portions (Figure 8C_1_, G_1_). One seventh to one fifth of the ostial part of the macula is not covered by the sagitta (Figure 8A, E). The ostial part of the macula sacculi not overlain by the sagitta is dorsally flanked by a distinctly thickened basal lamina in cave fish (Figure 4B_1_; see also Figure 10B) and in fish from the surface habitat Río Oxolotán (Figure 4C_1_). This thickening, however, is absent in surface fish from Tampico (Figure 4D_1_). The caudal sulcus impression—distinctly developed in surface fish—is filled with the otolithic membrane, but does not show a corresponding structure in the macula (Figure 8F *vs.* G_1_).

3D reconstructions suggest that a thick sagitta provokes a strongly curved macula sacculi (Figure 9D) whereas a thin sagitta comes along with a rather flat macula sacculi (Figure 9B). In our study, the sagitta of the individual of the surface ecotype was rather thick, which is only rarely seen in this ecotype [24], whereas the cave fish sagitta was comparatively flat explaining the curvature of the respective macula.

The maculae sacculi shows the four quadrant pattern of ciliary bundle orientation (Figure 8C_2_, G_2_; for number ciliary bundles on the macula see Table 2). In the caudal and central part, ciliary bundles of the ventral portion are oriented in ventral direction, while those in the dorsal portion are pointing dorsally. In the region of the dorsal and ventral bulges, the orientation gradually changes from a vertical to a horizontal orientation, resulting in dorsal ciliary bundles pointing caudally and ventral ciliary bundles being oriented in rostral direction in the ostial part of the macula. It is noteworthy that the dividing line also shows a dorsal ‘hump’ in the region of the dorsal bulge (Figure 8C_2_, G_2_). At its margins, the macula sacculi is flanked by a narrow band of ciliary bundles showing a kinocilium (4–5 µm) that is at least three times longer than the longest stereocilium (Figure 7C). Moreover, ciliary bundles with short kinocilia (∼2 µm) (Figure 7B) and ciliary bundles with kinocilia (3–4 µm) twice as long as the longest stereocilia (Figure 7A) are found throughout the macula. At the rostral margin, ciliary bundles forming an even staircase array with a kinocilium (3–4 µm) only slightly longer than the longest stereocilium can be observed (Figure 7A).

#### *Macula utriculi* (*N* = 4)

The maculae utriculi of both ecotypes are bowl shaped and have a short laterally positioned lacinia (Figure 10C). The lapillus covers the caudal portion (cotillus) of the macula, whereas the striola region and the lacinia are covered by otolithic membrane only (Figure 10A, D). The macula utriculi displays (i) oppositely oriented ciliary bundles in the striola region in the rostral portion of the macula (Figure 10E), (ii) medially and laterally oriented ciliary bundles in the lacinia region (Figure 10F), and (iii) ciliary bundles with rostral, rostro-lateral or rostro-medial orientation in the region of the cotillus (Figure 10E). In the striola region, ciliary bundles with a rather long kinocilium (4–5 µm) but forming an even slope (Figure 7A) are found near the dividing line. At the margin, mainly ciliary bundles with a kinocilium (∼3 µm) twice as long as the longest stereocilium can be seen (Figure 7A). The cotillus is dominated by (i) ciliary bundles forming an even slope and with a short kinocilium (Figure 7B) and (ii) ciliary bundles with a kinocilium twice as long as the longest stereocilium (Figure 7A). The lacinia is characterized by ciliary bundles with a long kinocilium (7–8 µm) of more than three times the length of the longest stereocilium (cf. Figure 7C; similar to the cb-type in ref. [6], Figure 5B).

No macula neglecta was observed in any of the studied specimens.

## Discussion

The inner ear of Atlantic mollies (*Poecilia mexicana*) displays a combination of several remarkable features. The utricle is connected rostrally instead of dorso-rostrally to the saccule, and the macula sacculi, therefore, is very close to the utricle. Moreover, the macula sacculi possesses a dorsal and ventral bulge where the orientation of ciliary bundles changes gradually. Interestingly, this region is also the transition zone where the macula is covered by the otolith on the one side while on the other (ostial-most) side it is covered by the otolithic membrane only.

### Remarkable features of the inner ear of *Poecilia mexicana*

Unlike in many other teleosts, the utricle of *P. mexicana* is connected rostrally to the saccule instead of dorso-rostrally. This position of the utricle and especially the vicinity of saccule and utricle (and thus of the two maculae, see Figure 10B–C) was so far only reported for medaka, *Oryzias latipes* ([25]: Figure 2A) belonging to the family Adrianichthyidae (Beloniformes) which is thought to be closely related to the members of the order Cyprinodontiformes [26]. It can be predicted that all or at least most cyprinodontiform fishes posses a similar inner ear morphology (cf. AN Popper, pers. comm.).

Though a similar rostral connection of the utricle to the saccule was reported from the short-snouted seahorse, *Hippocampus hippocampus* and the broad-nosed pipefish, *Siphonostoma typhle* (both members of the order Syngnathiformes) [27], the inner ears of these two species differ tremendously with respect to the proportions of the semicircular canals and end organs from those in *P. mexicana* and *O. latipes*.

The most prominent features of the macula sacculi of *P. mexicana* are the dorsal and ventral bulges. A dorsal and ventral bulge thus far has only been described for one gobiid species, the eye-bar goby, *Gnatholepis anjerensis* ([4]: Figure 7). A dorsal bulge was reported from five species belonging to different orders: dusky frillgoby, *Bathygobius fuscus* ([4]: Figure 7) (Gobiidae), Pacific fat sleeper, *Dormitator latifrons* [28] (Eleotridae), bonito, *Katsuwonus pelamis* ([4]: Figure 7) (Scombridae) (all Perciformes), oyster toadfish, *Opsanus tau* ([29]: Figure 2) (Batrachoidae, Batrachoidiformes), and redtail splitfin, *Xenotoca eiseni* ([3]: Figure 20) (Goodeidae, Cyprinodontiformes). In all aforementioned species, the orientation patterns of ciliary bundles in the macula sacculi change gradually within the region of the bulges just like in *P. mexicana*. With the exception of the cyprinodontiform species *X. eiseni* [3] and *P. mexicana* (this study), however, the sagitta in all other species covers the macula entirely ([4],[30]: Figure 1B). Although *X. eiseni* and *P. mexicana* share certain features (such as parts of the macula sacculi not being covered by the otolith, as well as the dorsal bulge), the macula sacculi of *X. eiseni* is straight, without a caudal or ostial widening. A differentiation (widening) of cauda and ostium of the macula sacculi in two species of hake, genus *Merluccius* (Merlucciidae, Gadiformes), was interpreted in terms of a regionalization (and thus, potential improvement) of frequency discrimination [31].

The sulcus acusticus of sagittae from other poeciliid taxa, like Perugia's limia, *Limia perugiae*, southern platyfish, *Xiphophorus maculatus*, and porthole livebearer, *Poeciliopsis gracilis*, shows prominent corresponding structures that may house the dorsal and ventral bulges of the macula sacculi ([9]: Figure 1a; T. Schulz-Mirbach, unpublished data). This may allude to the presence of well-developed bulges in poeciliids in general. The potential function of these bulges, however, is still entirely unknown.

The four quadrant pattern found in the macula sacculi of *P. mexicana* is typical for fishes without hearing specializations [2]. Thus, our results contrast with findings of Hertwig and Schneider [10] who reported on a ‘vertical’ pattern in the closely related guppy (*P. reticulata*). Such a vertical pattern, i.e. ciliary bundles being arranged in only two different orientation groups on the macula sacculi, is otherwise found only in otophysans and mormyrids [4], [32]. Therefore, the orientation pattern of the macula sacculi of *P. reticulata* should be reexamined. If the vertical pattern could be verified this could mean that *P. mexicana* and *P. reticulata* differ in their hearing abilities. Ibsch et al. ([9]: Figure 2a) examined the macula sacculi of the poeciliid *X. maculatus* and identified the same ciliary bundle types we detected in *P. mexicana*, but unfortunately, orientation patterns on the maculae were not described.

The macula lagenae of *P. mexicana* reveals the ‘crescent’ pattern as described by Popper and Coombs ([33]: Figure 4C) and displays an orientation pattern often found in teleost fishes without hearing specializations [33], [34]. Moreover, the orientation pattern of ciliary bundles of the macula utriculi is very similar to that seen in most teleosts [35].

Otoliths are about three times denser than the fish's body and thus, than the sensory epithelia. In consequence, the otolith lags behind the motion of the sensory epithelium and results in bending of the ciliary bundles of the sensory hair cells. This relative (microscopic) motion between otolith and sensory epithelium is either caused by swimming (linear or vertical acceleration) or sound stimulation in terms of particle motion [21], [36], [37]. Like *P. mexicana* and *X. eiseni*, there are several species that reveal maculae sacculi and/or maculae lagenae partly not covered by the respective otolith, e.g., yellow perch, *Perca flavescens* [3] (Percidae), lake whitefish, *Coregonus clupeaformis* [19] (Salmonidae), as well as some deep-sea fishes [5], [6]. According to the ‘quadrupole model’, uncovered hair cells should work as lateral quadrupole sensors, while hair cells that are covered by the otolith may act as dipole sensors [38]. Rogers and Zeddies [38] further proposed that this combination of dipole and quadrupole sensors might help the fish determine from which direction a sound emanates. Thus, beside the four orientation groups of ciliary bundles in the macula sacculi seen in the Atlantic molly, hair cells with horizontally oriented ciliary bundles can also be split into two subgroups (i.e. with and without otolith coverage) and could further increase the diversity of possible stimulation, which may enable the fish to precisely localize sound sources (cf. [38]).

### Methodological aspects

The study by Bang et al. [39] is one of only very few papers that show three-dimensionally reconstructed inner ears and maculae (see also [40]). Since then, software for 3D reconstruction of histological serial sections has improved tremendously (e.g., [41]). Our 3D models of maculae, for the first time, allow for a good estimation of the 3D curvature of sensory epithelia and hence the spatial arrangement of orientation groups of the ciliary bundles. This may be essential for the interpretation of the degree of variation in ciliary bundle orientation.

### Intra-specific variability

As our approach of combining various methods and also the 3D reconstruction were rather time-consuming, only a relatively small number of samples could be processed. Where we proceed to briefly discuss intra-specific variation, we will do so with the understanding that our approach precludes quantitative statistical analyses.

The stronger curvature of the macula due to thicker sagittae associated with a thicker otolithic membrane in the deep sulcus acusticus (a feature found in several cave fish specimens) may lead to a different spatial (i.e. more three-dimensional) arrangement of ciliary bundles. On a distinctly three-dimensionally curved macula sacculi displaying a four quadrant pattern of ciliary bundle orientation, it is likely that hair bundles not only show a morphological polarization in purely vertical or horizontal directions as on a rather flat macula, but there may in fact be more variation in ciliary bundle orientation according to the curvature of the macula in 3D space. This, in turn, may result in higher variation of possible ways of stimulating hair cells (see also below). Lu and Popper [28] demonstrated that the preferred directional response of a saccular ganglion neuron corresponds to the morphological polarization of hair cells innervated by that neuron.

For silver perch, *Bairdiella chrysoura* (Sciaenidae), it was hypothesized that the deep sulcus houses a thick otolithic membrane which may cause a modified transfer of shearing forces (see also [42]) to the ciliary bundles on the macula and thus, differences in hair cell stimulation [22]. In our study, we could show that the deep sulcus (mainly found in cave fish sagittae) indeed houses a tremendously thick otolithic membrane (OM), with a particularly thick and well developed upper part of the OM (for a detailed description of the components of the OM see [43]), while sagittae with a flat sulcus (often found in surface fish) house a thin otolithic membrane. Although it cannot be ruled out that the otolithic membrane was affected by tissue preparation procedures—especially by effects of tissue dehydration—it may be assumed that the observed differences in relative thickness of the otolithic membrane reflect an existing differentiation between ecotypes, as samples from both ecotypes were treated exactly the same way.

The sulcus acusticus in surface fish (Río Oxolotán) is rather narrow, which means that the region of intimate attachment between otolith and macula is small. It may be speculated that the caudal sulcus impression could serve as an additional attachment area for the otolithic membrane in order to better hold the sagitta in place. In cave fish the caudal sulcus impression is less developed than in surface fish (or even completely absent), especially in individuals with thick sagittae and deep sulci (for a quantification of these otolith features see [16], [24]). A deep sulcus, filled with a thick otolithic membrane, may result in a firm attachment of the sagitta to the macula sacculi, which, in turn, may compensate for the lack of a caudal sulcus impression.

Morphological follow-up studies on some of the apparently divergent traits uncovered here—using larger samples sizes—as well as neurophysiological studies are needed to test our hypotheses regarding potential differences in inner ear morphology between the cave- and surface-dwelling ecotypes.

### Conclusions

We suggest that other poeciliid taxa, or even other non-poeciliid cyprinodontiforms, may display similar inner ear morphologies as described here. Our detailed microanatomical investigation of the inner ear of *P. mexicana*, therefore, provides a basis for further research on structure-function relationships of inner ears within the species-rich order of toothcarps (Cyprinodontiformes), in particular in live-bearing species of the family Poeciliidae.

## Materials and Methods

### Study system and animals

*Poecilia mexicana* Steindachner, 1863 (Poeciliidae) is widespread in freshwater surface habitats along the Atlantic coast of Central America [44]. We focused on a cave population and two surface-dwelling populations of that species (Table 3). The cave form of *P. mexicana* (“cave molly”) originated from the sulfidic Cueva del Azufre in southern Mexico, a cave that is divided in 13 interconnected cave chambers [45]. A creek flows through the cave, forming several shallow pools that are partially divided by riffle passages. While the front cave chambers receive some dim light, the inner parts of the cave are lightless, and the molly population from the innermost cave chamber XIII [45] permanently lives in the dark. With the exception of chamber XIII, the water in all cave chambers is characterized by medium to high concentrations (up to 300 µM/L) of naturally occurring hydrogen sulfide [46], [47]. One of the surface populations originated from the Río Oxolotán, a river with sulfide-free water near the cave [46]. The second population of surface-dwelling fish came from brackish coastal waters near Tampico (Tamaulipas, eastern Mexico). Large, randomly outbred stocks of the cave and Río Oxolotán populations were maintained in 200-liter aquaria at the University of Potsdam since 2004; stocks from Tampico were founded using wild-caught fish in 1995. Cave and surface fish were kept under a 12∶12 h light∶dark cycle.

**Table 3:** Overview of *Poecilia mexicana* populations investigated, methods used, and number of specimens analyzed.

Population	Habitat type	Brain preparation/μ-CT of heads	Orientation patterns (SEM, CLSM)	3D reconstruction of end organs	Transversal semithin-sections of the saccule
Cueva del Azufre chamber XIII)	cave creek free of hydrogen sulfide; complete darkness	3/1	4	1	2
Río Oxolotán	sulfide-free river in the vicinity of the cave system; surface habitat	1/1	4	-	2
Tampico	brackish coastal waters; surface habitat	2/-	1	1	2

Investigation of the orientation patterns of ciliary bundles of sensory hair cells was performed using either a scanning electron microscope (SEM) or a confocal laser scanning microscope (CLSM).

Prior to any fixation and preparation, animals were anaesthetized and euthanized using an overdose of MS222 (SigmaAldrich).

### Gross morphology of brain and inner ears

Four specimens, i.e. two cave fish (one male, SL = 35 mm; one subadult individual, SL = 26 mm) and two fish from the surface habitat Tampico (two females, SL = 47 mm and 54 mm) were fixed in 10% aqueous formaldehyde solution for several months. The neurocranium was opened dorsally and bones and cartilage surrounding the brain and the inner ears were removed. Otoliths had been dissolved by the unbuffered formaldehyde solution. Images from ventral and dorsal views were taken using a Leica M165C stereomicroscope with a camera DFC 290 applying the multifocus option (extended focus imaging) of ImageAccess Standard 8 (Imagic AG, Glattbrugg, Switzerland).

### Position of otoliths *in situ*

In order to compare the orientation of otoliths to the main axis of the fish and also of the three different otoliths to one another, a micro-computed tomography (μ-CT) of one cave female (SL = 35 mm) and one surface (Río Oxolotán) female (SL = 38 mm) was performed by RJL Micro & Analytic GmbH (Karlsdorf-Neuthard, Germany) with a ScyScan 1172 at 100 kV. Specimens were preserved in 70% ethanol, and 3D data of each head were taken at a resolution of 5 µm per voxel (isotropic). The back-projection of the tilt series resulted in virtual tangential section series of 2,205 slices (cave fish) and 2,202 slices (fish from Río Oxolotán). After the X-ray analysis, otoliths were dissected, cleaned with 1% KOH solution and distilled water, and investigated using scanning electron microscopy (LEO 1430VP). Serial μ-CT-scans of otoliths and parts of the bones of each fish were three dimensionally reconstructed (after threshold-based segmentation of bony structures) using the VolumeRendering tool (threshold setting for all otolith types: 65) in AMIRA® v. 4.1.1 (Visage Imaging GmbH, Berlin, Germany). The threshold was determined according to the best concordance of reconstructed otoliths with the original ones represented by the SEM images. Subsequently, otoliths were separated from the ‘master’ LabelField file into six LabelFields, each containing a single otolith only and saved as separate files. The new LabelFields were reduced in resolution applying the Resample module. Surface rendering was performed with the SurfaceGen module. Then, the number of triangles of the generated SURF file was reduced with the Simplifier tool to as low as 249,972 (surface) or 99,996 (cave) for sagittae, 60,000 (surface) or 52,188 (cave) for asterisci, and 30,000 (surface) or 20,000 (cave) in the case of lapilli. This was followed by the smoothing of surfaces using the SmoothSurface module (mostly 20 iterations; constrained smoothing). Finally, otolith volumes and angles were measured applying the ‘Measure – TissueStatistics’ tool of AMIRA®.

In the case of angle measurements, all separated SURF files of the otoliths from each specimen were loaded into AMIRA®. Measurements of the angle between the sagitta and the main axis of the fish were performed in dorsal view. The sagitta and asteriscus were positioned in caudal view and the angle between the two otoliths was measured. The rotation of otoliths into the final position of measurement was performed twice with a precision of angle measurements to the nearest 1°.

### Sensory epithelia: orientation patterns of the ciliary bundles

We additionally analyzed the orientation patterns of ciliary bundles because—as in all vertebrates—ciliary bundles of hair cells of the end organs in teleosts are organized in different orientation groups on the sensory epithelium (e.g., [19], [48]). This was done by scanning electron microscopy as well as confocal microscopy after immunofluorescence labeling.

### Scanning electron microscopy

Five heads (cave fish: two males, SL = 30 and 31 mm; surface fish: Río Oxolotán: two females, SL = 34 and 40 mm; Tampico: one female, SL 36 mm; see Table 3) were cut medio-sagittally and fixed in (i) 2.5% glutaraldehyde solution, (ii) 4% formaldehyde solution in 0.1 M phosphate buffer overnight at 4°C, or (iii) in 5% glutaraldehyde and 1% osmium tetraoxide solution in 0.1 M phosphate buffer on ice for 4 h. Inner ears were then dissected and washed several times with 0.1 M phosphate buffer and postfixed with 1% osmium tetraoxide solution in 0.1 M phosphate buffer on ice for 1–2 h. Subsequently, otoliths were dissected and the otolithic membrane was removed. Then the maculae were dehydrated through a graded acetone series (30%, 50%, 70%, 80%, 90%, 96%, followed by 3 steps with 100% acetone) at 20 minutes intervals prior to critical point drying. For critical point drying carbon dioxide was used as intermediary fluid using a BAL-TEC CPD-030. The maculae were mounted on aluminum stubs medial-side up (macula sacculi and macula lagenae) or dorsal-side up (macula utriculi) with platelets of conductive glue or on conductive plasticine (Leit-C-Plast, Plano GmbH). The stubs were coated with gold using a Polaron SC510, and images were taken with a LEO VP1430 at 13 kV. Orientation of ciliary bundles and ciliary bundle types were identified using SEM images at magnifications of 2,000- to 3,000-fold.

### Immunocytochemistry

As an alternative method to the SEM investigation, ciliary bundles of the maculae of two males from the cave (SL = 30 and 31 mm) and two females from the surface habitat ‘Río Oxolotán’ (SL = 34 and 41 mm) were stained according to the method introduced by Lu and Popper [49] with TRITC-labeled phalloidin (Sigma-Aldrich, St. Louis, MO, U.S.A.) for stereocilia and anti-bovine α-tubulin mouse monoclonal antibodies (Molecular Probes®, Invitrogen, Darmstadt, Germany) and Alexa Fluor 488 conjugated anti-mouse secondary antibodies (Molecular Probes®, Eugene, OR, U.S.A.) for kinocilia. Nuclear counterstaining was done with TO-PRO-3 iodide (Molecular Probes®, Eugene, OR, U.S.A.). Prior to staining, heads were fixed for 15 minutes in 4% buffered (0.1 M phosphate buffer) formaldehyde solution at room temperature, then the skull roof was removed, heads were cut medio-sagittally and the brain was carefully removed, and samples were fixed for another 1.5 h at 4°C. During the fixation period, the fixative was exchanged once after 40 minutes. Then inner ears were dissected in fixative and otoliths removed. Inner ears were washed four times in 0.1 M phosphate buffer with 0.01% sodium azide at 20 minute intervals at room temperature on a slowly moving shaker. All further steps (unless specified otherwise) were performed at room temperature on a shaker, and after every staining/antibody step tissue samples were washed four times with phosphate buffer with sodium azide at 20 minute intervals. Inner ears were incubated in blocking solution for 1 h and then incubated overnight in anti-bovine α-tubulin mouse monoclonal antibodies (1∶200 dilution in 0.1 M phosphate buffer with sodium azide). Inner ears were incubated in Alexa Fluor 488 anti-mouse antibodies (1∶200 dilution in 0.1 M phosphate buffer) for 1.5 h at 37°C, in TRITC-labelled phalloidin (1∶100 dilution in 0.1 M phosphate buffer) for 4 to 5 h, and TO-PRO-3 iodide (1∶500 dilution with 0.1 M phosphate buffer) for 40 minutes. After the staining procedure, samples were stored at 4°C for one day, after which the maculae were mounted on a slide with an anti-fading medium, VectaShield® (Vector Laboratories). In this medium, the maculae were carefully flattened and then covered with a cover slip, sealed with nail polish, and stored at 4°C.

In order to estimate the overlap of the maculae and otoliths, saccular and lagenar otoliths from the respective maculae were cleaned with 1% KOH solution and distilled water, and investigated using a scanning electron microscope (LEO 1430VP).

### Confocal imaging

**Figure 11:**
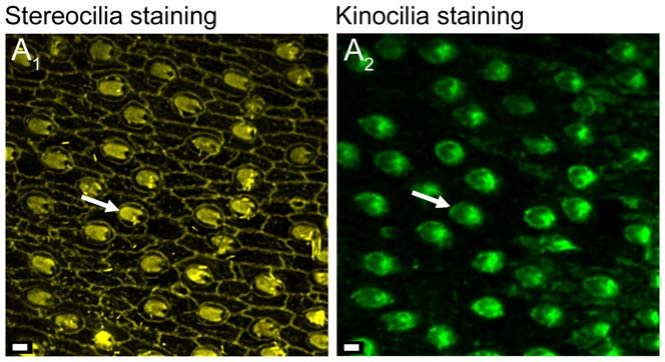
Confocal images of double labeling of a saccular epithelium. (**A_1_**), stereocilia of ciliary bundles stained with TRITC-labeled phalloidin. (**A_2_**), kinocilia of the same ciliary bundles stained with anti-bovine α-tubulin mouse monoclonal antibodies and Alexa Fluor 488 conjugated anti-mouse secondary antibodies. White arrows indicate the orientation of ciliary bundles based on the position of the kinocilium. Scale bars = 2 µm.

Samples were investigated with a Leica TCS SP5 confocal laser scanning microscope (CLSM) using a Leica HCX APO L U-V-I 40× long distance water dipping objective (NA = 0.8) and with the 488 nm argon-gas-laser line, the 561 nm diode pumped solid state laser, and the 633 nm He-Ne laser. The staining with TRITC-labeled phalloidin clearly showed the labeled stereocilia and a ‘hole’ for the non-labelled kinocilium (Figure 11A_1_), while kinocilium-staining (Alexa Flour 488) displayed the specific labeling of kinocilia only (Figure 11A_2_).

### Quantification of the number of ciliary bundles and the macula area

For the analysis of the maculae sacculi up to 34 overlapping image stacks (20 to 40 single images per stack) were photographed, in the case of the maculae lagenae up to 11 overlapping image stacks were taken (20 to 40 images per stack) at a z-step size of 500 nm and a pixel size of 250 nm×250 nm. The image stacks of each color channel were reduced to one image by applying the brightest point projection tool in Image J v. 1.43. Brightest point projected images were used for creating one map of the stained stereocilia and a second map of the stained kinocilia of the maculae in Adobe Photoshop CS2®. Orientations of ciliary bundles were determined as described by Lu and Popper [49] and ciliary bundles of the whole maculae were counted after dividing the map into up to 35 to 40 non-overlapping parts for the maculae sacculi and up to 10 parts for the maculae lagenae.

The area of the maculae sacculi and maculae lagenae was determined as follows: The outlines of the maps generated from the stained stereocilia were drawn in Adobe Illustrator CS2®. Then a BMP file was generated in Adobe Photoshop CS2® showing a white macula on a black background. These BMP files were loaded into the tpsDig2 software [50], and the area of each macula was determined applying the ‘ImageTools – Measure’ option. Finally, a ratio of the number of ciliary bundles and the respective macula area was calculated.

The percentage of the macula sacculi and macula lagenae not covered by the sagitta and asteriscus was calculated by dividing the non-covered area by the total macula area. The exact overlap was evaluated by bringing the SEM images of the medial face of the otolith and the outline of the respective macula (see procedure above) to the same scale and fitting the macula sacculi (or macula lagenae) into the sulcus acusticus of the sagitta or the fossa acustica of the asteriscus, respectively. Then, the part of the macula not covered by the otolith was outlined in Adobe Illustrator CS2® and the area determined as described above for the total macula area.

### 3D reconstruction of end organs

Light microscopical and histological procedures followed Ruthensteiner [51]. The right halves of the heads of two specimens (one cave fish male, SL = 35 mm; one surface fish female from ‘Tampico’, SL = 52 mm) were fixed in 2.5% glutaraldehyde solution in 0.1 M phosphate buffer overnight at 4°C, washed three times in 0.1 M phosphate buffer, and stored at 4°C until further processing. The labyrinth was dissected and decalcified in 2% ascorbic acid for 5 days at room temperature, washed several times in 0.1 M phosphate buffer, postfixed in 1% osmium tetraoxide solution in 0.1 M phosphate buffer on ice for 1 h, washed in 0.1 M phosphate buffer, dehydrated in a graded acetone series and embedded in Epon (Carl Roth GmbH & Co. KG, Karlsruhe, Germany). Serial sectioning (sections à 1 µm) was performed with a ‘Histo Jumbo’ diamond knife (Diatome AG, Biel, Switzerland) on a RMC-MT XL ultramicrotome. Semithin-sections were stained with Richardson's solution [52]. The macula, the non-sensory epithelium, and the basal lamina of the end organs of the cave fish (transversal sections; saccule 427 sections, lagena 205 sections, and utricle 25 sections) and of the surface fish (sagittal sections; saccule 380 sections, lagena 129 sections) were three-dimensionally reconstructed using AMIRA® v. 4.0.1 (Visage Imaging GmbH, Berlin, Germany). In part, the otolithic membrane, remains of the otolith, and parts of the eighth cranial nerve were reconstructed as well. For the reconstruction every second section of the series was photographed with a Leica DFC 480 microscope camera on a Leica DMLB microscope (Leica Microsystems, Wetzlar, Germany) at an image resolution of 1,280×960 pixels (object lens: 20×; resolution: 0.5 µm/px). Section images were saved at 24 bit RGB color depth in TIF format. They were subsequently contrast enhanced, unsharp masked, and color format was changed to grayscale in Adobe Photoshop CS2®. In the case of the saccules of both specimens and the utricle of the cave fish, pixel resolution of images was reduced to 0.8 µm/px. Slice alignment and 3D surface rendering was performed using AMIRA® software. Slice alignment was performed using the alignment tool (least squares method) and if necessary was corrected manually by bringing the structures of neighboring slices to a maximum congruence. Labeling of structures (AMIRA: ‘segmentation’) was done manually using the brush tool. Generally, every slice was labeled. Whenever possible, however, only every third to fifth slice was used for the 3D reconstructions, with subsequent interpolation of structures on intervening slices, followed by subsequent check and—if required—correction of segmentation results. Surface rendering was mainly performed as described in ‘Position of otoliths *in situ*’. Surfaces were smoothed using the SmoothSurface module (unconstrained smoothing and 40 iterations).

### Contact region: sensory epithelium – otolithic membrane – otolith

In order to investigate the effects of sulcus depth on the sensory epithelium and the otolithic membrane, transversal sections of the saccule of two additional cave individuals (one female, SL = 51 mm; one male, SL = 37), one surface female from Río Oxolotán (SL = 44 mm) and one surface fish from Tampico (female, SL = 30 mm) were studied. In addition, the sections were compared with SEM-based surface images of the corresponding left otoliths. Samples were fixed, embedded, cut, and stained as described in the previous paragraph.
